# Migration and health in Southern Africa: 100 years and still circulating

**DOI:** 10.1080/21642850.2013.866898

**Published:** 2014-01-02

**Authors:** Mark N. Lurie, Brian G. Williams

**Affiliations:** ^a^ Department of Epidemiology, Brown University School of Public Health, Providence, RI, USA; ^b^ South African Centre for Epidemiological Modelling and Analysis, University of Stellenbosch, Stellenbosch, South Africa

**Keywords:** migration, health, HIV, TB, Africa

## Abstract

Migration has deep historical roots in South and Southern Africa and to this day continues to be highly prevalent and a major factor shaping South African society and health. In this paper we examine the role of migration in the spread of two diseases nearly 100 years apart: tuberculosis following the discovery of gold in 1886 and HIV in the early 1990s. Both cases demonstrate the critical role played by human migration in the transmission and subsequent dissemination of these diseases to rural areas. In both cases, migration acts to assemble in one high-risk environment thousands of young men highly susceptible to new diseases. With poor living and working conditions, these migration destinations act as hot-spots for disease transmission. Migration of workers back to rural areas then serves as a highly efficient means of disseminating these diseases to rural populations. We conclude by raising some more recent questions examining the current role of migration in Southern Africa.

## Introduction

Population movement, or human migration, has historically played a critical role in the spread of disease globally. Early explorers like Columbus arrived at distant shores not only armed, but also infected with syphilis and other diseases which were then easily passed to local populations who had never previously been exposed to such diseases. More recently, the rapid and global spread of severe acute respiratory syndrome (SARS) in 2003–2004 clearly illustrates the impact of human movement on disease dissemination: first detected in southern China in November 2003, within three months 305 cases were detected in a neighbouring state. By the next month, nine other countries were reporting SARS cases, and six months later it had become a global epidemic with 33 countries reporting over 8000 cases and a case fatality rate of just below 10% (WHO, 2003). Later phylogenetic analysis of the virus found a high probability that the SARS coronavirus originated in bats and spread to humans, either directly, or through animals kept in Chinese markets (Li et al., 2005).

The SARS example illustrates clearly how, in a highly interconnected world where an infectious person can board an aeroplane and be halfway around the world before the end of the infectious period, the movement of people is critical to the spread of disease. The case also illustrates how transmission often occurs in “hot-spots” (in the case of SARS, in places where humans and animals had maximum contact) and then follows the movement of infected individuals, who subsequently infect people in the new places to which they migrate.

A 2007 case further illustrates the point that global migration and interconnections can fuel the spread of disease, not to mention panic. In 2007, a man infected with multi-drug-resistant tuberculosis, flew from Atlanta, Georgia to France and on to Greece and Italy, then returned on a flight from Prague, Czech Republic to Montreal, Canada where he crossed over the border back to the USA. The US Centers for Disease Control and Prevention (CDC) believed that he was suffering from extensively drug-resistant TB and a major alert was raised while the authorities attempted to find him. When he returned to the USA, he was placed under involuntary isolation, becoming the first person in the USA since 1963 to be subject to CDC isolation under the Public Health Service Act of 1944.

Migration has become one of the most important determinants of global health and social development (Carballo, Divino, & Zeric, 1998; Quinn, 1994). People are moving in greater numbers and over larger distances than ever before, and migration has important implications for those who migrate, those who are left behind, and those communities that host migrants.

Migration can act to speed the transmission of an infectious disease in one of two ways: it can act as a bridge between geographical areas, essentially linking high and lower prevalence areas trough the movement of infected people. A second way migration can act to speed transmission is if migration induces increased risk behaviour. For tuberculosis, for example, in Southern Africa, as we will see, migration not only links high transmission urban areas to previously uninfected rural ones, but it also exposes migrants to significantly higher risk at the workplace through factors like unclean working conditions, exposure to silica dust and overcrowded housing (McCulloch, 2012; Stuckler, Basu, McKee, & Lurie, 2011). A variety of additional theoretical frameworks have been proposed, but are beyond the scope of this paper (see, for example, Crush, Williams, Gouws, & Lurie, 2005; Deane, Parkhurst, & Johnston, 2010; Hirsch, 2013; Weine & Kashuba, 2012).

In this paper we examine the role of migration in the spread of two diseases in Southern Africa nearly 100 years apart: TB in the early part of the 1900s and HIV since the 1990s. Both examples demonstrate clearly the critical role played by human migration in the dissemination of these diseases, first from transmission “hot-spots” and then to rural areas when migrants migrated back home. We conclude the paper by discussing some new questions relating to migration and health in the context of a changing HIV/AIDS epidemic and expanded access to treatment.

## Changing migration patterns in Southern Africa

The example of SARS sheds light on the processes of human infection and migration, and in Southern Africa the spread of HIV and TB fit clearly into this pattern. Gold, discovered in South Africa in the late 1800s, fuelled a mass migration of young men from rural areas to work, temporarily, on the mines. They were labelled “temporary workers” because the South African government worked hand-in-hand with mining authorities to devise a system to bring young black men from rural areas to work on the mines. But permanent residence on the mines was prohibited as the government sought to expand the separation of races (later codified and formalized as *apartheid*), while the mining companies sought to establish a system of migrant labour which would provide them with an adequate supply of workers. Furthermore, government and mining officials conspired to establish the system of circular of oscillating migration – in which men work on the mines for fixed periods, and then return home to their rural “homelands”. By design, the system was established to ensure the adequate supply of labour to the mines and also to absolve the mining companies of the long-term care of former miners (Lurie, 1992). By sending miners home after the conclusion of their work contracts, the mining companies conveniently externalized the long-term costs of caring for these often sick former workers, many of whom had contracted TB and silicosis on the mines during the periods of employment.

Since whites reserved the most productive land for themselves, the rural “homelands” were for the most part highly impoverished areas (Palmer & Parsons, 1977). Furthermore, the imposition of the “hut-tax”, beginning in 1884, forced many young men to go to the mines because they needed money to pay the tax. As a result, millions of people were forced to migrate temporarily to “white areas” to work in the mines, industry, agriculture and in the domestic labour sectors. Permanent migration to “whites-only” areas was strictly prohibited under apartheid laws, and even temporary entrance into “whites-only” areas necessitated a stamp in ones ID or “passbook”.

By allowing black people to live only temporarily in “white areas” these and other laws sought to ensure that black South Africans retained a rural base (Packard, 1989), an essential element in the financial structure of the mining industry and of apartheid itself. The fact that blacks supposedly had access to rural land was a continuous justification used by the mining industry to pay low wages to its workers.

The changing and dynamic nature of migration in Southern Africa can best be summed up by examining rural livelihoods in the early and latter parts of the twentieth century. In the mid-1930s, between 40% and 50% of rural subsistence food requirements were produced in the rural areas (May, 1990). By 1970, agricultural production in rural areas had declined to such an extent that only 10% of the total income for the majority of rural households came from agriculture (May, 1990) and rural residents had become increasingly dependent on remittances from their family and relatives working in urban areas. The majority of a rural households' income came instead from remittances by family members employed as migrant labour and in KwaZulu/Natal, for example, by the late 1980s remittances made up over three-quarters of rural household income (May, 1990).

Migration in South Africa is not static but has changed considerably over the last three decades (Crush & James, 1995) and further dramatic changes in migration patterns have occurred following the democratic transition in 1994. For at least the first half of the 1900s, migration for most workers was temporary, and people tended to work in the mines or other sectors for a few years and then return permanently to the rural “homelands”. In 1936, for example, there was a greater than 75% chance that by the time a migrant was 45 years old, he would no longer be a migrant labourer, and instead would be living in a rural “homeland” (Natrass, 1976).

The character of migration to the gold mines has also changed considerably over the last two decades. Whereas in the early 1970s almost 80% of the work force on the gold mines came from outside South Africa, today less than 40% of gold miners come from outside South Africa's borders (Crush & James, 1995). Thus migration acts as a link between all countries of Southern Africa.

In addition, large-scale retrenchment over the last decade has come about as a result of falling levels of production, a dramatic drop in the international price of gold and the historical marginality of South Africa's gold mines. For example, in 1992 the gold mines employed 32% fewer workers than they did in 1986. Foreign miners are less likely to be laid off than South Africans – between 1986 and 1992, 36% of South African miners were laid off; at the same time, 27% of foreign miners were retrenched (Crush & James, 1995).

Despite these changes, important aspects of mine migration have persisted: over 90% of the industry's black employees are migrants, and the vast majority live in single-sex hostels and visit their rural families only occasionally (Crush & James, 1995). Foreign workers on the mines are generally able to return home less frequently than their South African counterparts, and their partners are less likely to be able to visit them on the mines.

In reality, little has changed in terms of the living arrangements for the majority of miners over the past decade. In 1993, 89% of miners lived in single-sex hostels (Crush & James, 1995). That number is slowly declining as the mining industry experiments with the introduction of married housing. Still, in 1993, only 2.1% of gold miners lived in married housing (Crush & James, 1995); that number has changed little in the last two decades.

One thing that has changed considerably since the early 1990s is the ability of people to move easier, more frequently and over longer distances. With the demise of apartheid and restrictive laws that tightly controlled people's movements, as well as more flexible work contracts and improved transportation infrastructure, migrants are now able to return home much more frequently that they were in the past.

But patterns of migration have changed considerably in South Africa in the last two decades, particularly following the abolition of apartheid and the first democratic election in 1994 (Posel, 2006). These changes are characterized by both an increase in the prevalence of female migration (Posel, 2006; Posel & Casale, 2003) and an increasing frequency with which migrants are able to return home (Crush, 1992). Prior to the 1970s, most work contracts on the gold mines were for six-month duration; over the following two decades, most labour contracts on the mines were lengthened and became highly stabilized (Crush, 1992). For example, in 1981, 59% of labour contracts on the gold mines were for 45 weeks and 19% were for 52 weeks. By 1987, 90% of labour contracts were for 52 weeks with only 7% being 45 weeks. As a result, net labour turnover on the mines decreased significantly, indicating longer work-stays, reduced turnover and greater workforce stability (Crush, 1992).

These changes have resulted in an increase in labour and other migration as well as more frequent visits home by migrants. The impact of this movement if obvious in terms of TB – more frequent returns home render rural families more vulnerable since people with a higher likelihood of infection are spending more time in these rural communities and therefore exposing more people to potential infection. Little is known, however, about the impact of increased frequency of returning home on HIV transmission, and increased frequency of return in this example could potentially be protective: if migrants return home more frequently, they are perhaps less likely to take additional sexual partners while away; conversely, the increased frequency of return could result in more frequent exposure of uninfected partners.

Despite these changes, the prevalence of migration remains extremely high. In rural KwaZulu/Natal nearly 60% of adult men spend most nights away (Lurie, Harrison, Wilkinson, & Abdool Karim, 1997) and this number is similar in other rural areas of South Africa (Collinson et al., 2006).

## Migration and the spread of tuberculosis

Packard (1989), in his seminal book *White plague, black labour: Tuberculosis and the political economy of health and disease in South Africa*, traces the spread of TB in South Africa following the establishment of mining camps on the South African Witwatersrand. Using historical data, Packard illustrates clearly how mining camps became a “hot-spot” of TB transmission and how the system of circular migration then resulted in infected miners returning home to infect their partners and families. That the mining camps were an early hot-spot for TB transmission should come as no surprise as the mines contained a lethal mix of conditions highly conducive to the spread of disease: lungs weakened by exposure to silica dust (which causes silicosis and renders people highly susceptible to TB), long hours working underground in extreme heat and under dusty conditions, and crowded housing arrangements that often saw more than 16 people sharing the same room.

That TB then spread from these urban hot-spots to rural areas should also come as no surprise: hundreds of thousands of men, living and working in conditions conducive to the spread of TB, were released on an annual basis to return home, where they often unwittingly exposed partners, family and community members to a disease that, until that time, had not yet been detected in rural areas. In addition, the policy of repatriating sick workers to rural areas “guaranteed that the epidemic of urban-based TB quickly spread to the rural hinterlands” (Packard, 1989) so that by the late 1920s, as many as 90% of the adult population living in the Transkei and Ciskei had been infected with TB. The oscillatory migration then served as a perfect vehicle for disease dissemination, with migrant miners acting as the “vector” bringing home new, hitherto unseen, diseases.

Migrants may act as “bridging” populations as they are usually more at risk of infection both because of their working conditions and also because they are living in areas with higher background prevalence, increasing the chances that they are exposed to an infected individual (Coffee, Lurie, & Garnett, 2007; Lurie et al., 2003). Thus migrants return with a higher prevalence of disease, thereby unwittingly exposing their partners, families and communities to an increasing risk of infection.

## Migration and HIV/AIDS

The emergence of HIV/AIDS nearly a century later followed a similar path. Over the intervening century, the separation of races became codified in South African law, and the system of circular migration became even more deeply entrenched. Myriad laws were created to ensure that black South Africans would not remain in “whites-only” areas, formalizing the system of oscillating migration where employed migrant men returned annually to rural areas and then more permanently as they became too old or too sick to work.

By the mid-1980s, South African mines employed roughly a million people, nearly half of whom were migrants from within South Africa's borders with the other half coming from about a dozen countries in Southern Africa including Lesotho, Swaziland, Botswana and Zimbabwe. In Swaziland by the early 1990s, migration to the mines accounted for more than 16% of the country's formal employment and 36% of households had at least one member working in South Africa, the vast majority of whom worked in the mining industry (Leliveld, 1997). And while the mines were among the largest employers, other industries attracted temporary, migrant workers in much the same way.

At the very time that HIV/AIDS was becoming established in West and East Africa, South Africa was in the midst of its own largely peaceful transition, and with the release of Nelson Mandela and the unbanning of political parties in 1990, followed by the first democratic election in 1992, the new government had its hands full writing a new constitution, establishing a supreme court and re-writing laws that for a century had been designed to maintain white power and privilege. Fully occupied with these matters, the government was slow to respond to HIV/AIDS, and when it did, it often did so in a way that was not helpful, and frequently detrimental, to prevention (Kalichman, 2009).

Ironically, the lifting of apartheid laws and the advent of democracy may inadvertently have helped fuel the spread of HIV by facilitating the movement of people both within the country and from other countries in the region. The patterns of migration that were established by the middle of the twentieth century was one of oscillating migration where migrant men returned home at the end of every year. This pattern of annual return began to change in the 1970s with improved transportation infrastructure and the growth of powerful trade unions which over the years were able to negotiate more flexible work contracts.

Patterns of migration continued to change considerably in South Africa in the last two decades, particularly following the abolition of apartheid and the first democratic election in 1994 (Posel, 2006). The migration of women has increased (Posel, 2006; Posel & Casale, 2003) and migrants are able to return home more frequently (Crush, 1992). Before the 1970s, most work contracts on the gold mines were for six-month duration but over the following two decades they were lengthened and became highly stabilized (Crush, 1992). For example, in 1981, 59% of labour contracts on the gold mines were for 45 weeks and 19% were for 52 weeks. By 1987, 90% of labour contracts were for 52 weeks with only 7% being 45 weeks. As a result, net labour turnover on the mines decreased significantly, indicating longer work-stays, reduced turnover and greater workforce stability (Crush, 1992).

Ironically the end of apartheid has the effect of making the population even more mobile, allowing people to return home more frequently. And this more frequent return also means more frequent exposure to infectious diseases.

While urbanization has been happening, a persistent trend has been the maintenance of family ties in rural areas, so that many migrants to South Africa's urban centres consider themselves temporary migrants who will return to rural areas at the end of their migration. Some, in fact, are returning to die in rural areas, further demonstrating the strong social and familial ties to rural areas (Clark, Collinson, Kahn, Drullinger, & Tollman, 2007).

## New questions about migration and disease: from migration-induced disease to disease-induced migration

Early studies of migration and health in Southern Africa focused on migration as a vector for disease spread. Studies sought to show how migrants were at increased risk for contracting TB and HIV at their work places, and how their return to rural areas exposed their families and partners to diseases contracted on the mines. We thus focused on *migration-induced disease*: the additional health risks migrants were exposed to as a result of their migration, followed by the additional risk that partners (and families) of migrants were exposed to when the migrant returned home.

Today, while migration-induced disease is still important, new aspects of migration and health are emerging that requires additional study. Southern Africa, now in the midst of the epidemiological transition, faces simultaneous epidemics of infectious and chronic diseases. What these infectious and non-infectious agents have in common is the need for long-term – perhaps lifetime – chronic care. For people with HIV/AIDS, taking lifesaving antiretroviral drugs is a daily event that should continue over the lifetime. Similarly, the treatment of obesity, diabetes and other chronic infections now becoming common in Southern Africa requires lifelong treatment. Yet, in the context of the continued large-scale human migration, important questions are now being raised about how people who are so mobile will be able to maintain lifelong treatment for these chronic infections. How do migrants negotiate access to care, for example, after the leave urban employment? How do people who migrate frequently negotiate daily pill regimens, and how and where do they decide to get treated. Do migrants prefer to access treatment for some diseases in rural areas, while for other diseases in urban areas? These and other related questions about the role of migration in accessing and negotiating treatment are among the new important questions raised by migration and health today. While we must still be interested in migration-induced disease – and in finding ways to mitigate the additional health risks of migration, we must increasingly turn our attention to *disease-induced migration*, where sick people may change their patters of migration in order to access quality care.
